# Sharing a medical decision

**DOI:** 10.1007/s11019-023-10179-3

**Published:** 2023-11-27

**Authors:** Coos Engelsma

**Affiliations:** Department of Ethics, Centre for Dentistry and Oral Hygiene, University Medical Centre Groningen, University of Groningen, Groningen, The Netherlands

**Keywords:** Shared decision making (SDM), Patient autonomy, Paternalism, Preference rankings

## Abstract

During the last decades, shared decision making (SDM) has become a very popular model for the physician-patient relationship. SDM can refer to a process (making a decision *in a shared way*) and a product (making a *shared decision*). In the literature, by far most attention is devoted to the process. In this paper, I investigate the product, wondering what is involved by a medical decision being shared. I argue that the degree to which a decision to implement a medical alternative is shared should be determined by taking into account six considerations: (i) how the physician and the patient rank that alternative, (ii) the individual preference scores the physician and the patient (would) assign to that alternative, (iii) the similarity of the preference scores, (iv) the similarity of the rankings, (v) the total concession size, and (vi) the similarity of the concession sizes. I explain why shared medical decisions are valuable, and sketch implications of the analysis for the physician-patient relationship.

## Introduction

During the last decades, shared decision making (SDM) has become a very popular model for the physician-patient relationship. Instead of the physician having full authority to decide on a treatment plan, or the patient having all power to determine a course of action, the leading thought has become that the physician and the patient should ideally arrive at a treatment plan together (e.g. Veatch 1972; Emanuel and Emanuel 1992; Charles et al. 1997; Elwyn 2021). One feature of SDM, only occasionally mentioned in the literature, is that it may refer to two different things, both capable of being shared: a *process* and a *product*. SDM is naturally construed as referring to the process of forming or working towards a decision: the steps the physician and the patient undertake in their attempt to reach a decision together (e.g., Stiggelbout et al. 2015; Bomhof-Roordink et al. 2019). This construal emphasizes the *shared making* of a decision. However, SDM can also be taken to refer to the product of that process: a decision finally made. The latter construal stresses the making of a *shared decision*. As Charles et al. say,

[s]hared decision-making is usually depicted, either implicitly or explicitly as a type of decision-making process. But shared treatment decision-making can also refer to an outcome, i.e. a shared or agreed upon decision (Charles et al. 1997, 688).^1^

That SDM can refer to either a process or a product is also reflected in the language used by SDM researchers. Many of them talk about a ‘process’ of shared decision making:

The patient’s participation in clinical decisions is fostered by the legal doctrines of consent and informed consent and by the ethical process of shared decision making (Whitney et al. 2003, 54).

Shared decision-making (SDM) is defined as a decision making process jointly shared by patients and their health care providers (Gravel et al. 2006, 2).^2^

Others talk about a ‘decision’ that has to be shared:

[C]linicians (…) have an ethical imperative to share important decisions with patients (Salzburg Global Seminar 2011).

This SDM framework was developed in order to allow for a more comprehensive and expansive understanding and analysis of how decisions are shared in clinical practice (Callon et al. 2018, 1575).^3^

Given this distinction, the obvious question is how the process and the product are related. One possibility is that the two depend on each other, e.g. such that a shared decision simply *is* a decision reached through a shared process, or such that a shared process simply is a process leading to a shared decision.

Though the option of defining a shared decision in terms of a shared process, or a shared process in terms of a shared decision, would simplify matters, doing so would fail to do justice to the concepts at issue. It is possible that an unshared process can lead to a shared decision. For instance, a paternalistic process in which only the physician provides information about the disease and treatment options, adduces arguments, proposes a choice, etc., may still lead to a decision with which the patient fully agrees (cf. Charles et al. 1997, 688). Also, it is imaginable that a shared process leads to a decision that is not shared or only minimally shared. Even if a physician and a patient collaborate very constructively in order to jointly reach a decision, their attempt may fail, or succeed only in some minimal sense. The process may end with a decision that one of the parties regards far from optimal, or it may result in no decision at all (or a decision to ‘agree to disagree’). As Charles et al. note,

[a]greement between physician and patient about the treatment decision is one possible outcome of this [shared] process; others include no decision or disagreement as to the preferred treatment (1997, 688).^4^

In the literature on SDM, much attention is devoted to the decision making process: what should be done by the physician and the patient in order for them to jointly reach a treatment decision. The steps in this process have been categorized in various ways. One influential way is provided in the classical account of Charles et al. (1999).^5^ On that account, every (medical) decision making process involves three distinct stages: (i) the *information exchange stage*, where information is given that is deemed relevant for the treatment decision; (ii) the *deliberation stage*, in which treatment preferences are being considered, weighed, and discussed; and (iii) the *decision stage*, where the decision is actually made (ibid., 654-8).

Literature on the information exchange focuses on what the physician should tell the patient, e.g. about his disease and about available treatment options and their benefits and risks; and also on what the patient should tell (or should be facilitated or empowered to tell) the physician, e.g. about his personal circumstances, values and goals, desires and fears, etc. (ibid., 654-6; Wirtz et al. 2006, 119–121; Stiggelbout et al. 2015, 1174–1175; Entwistle et al. 2011).^6^ Literature on the deliberation stage addresses questions concerning how the physician and the patient should, or should be allowed, to deliberate within SDM. Presumably, both should express their preference for a treatment alternative. Yet should they also discuss these alternatives, exchanging arguments for and against them, if their initial preferences differ? May the physician (or the patient) employ rhetorical means to persuade the patient (the physician), and is it allowed to negotiate about treatment alternatives (Charles et al. 1999, 656-8; Wirtz et al. 2006, 121-2; Sandman 2009; Sandman and Munthe 2010, 74 − 8)? Literature on the decision making stage focuses on the question who has decisional authority within SDM. For instance, is SDM compatible with a physician proposing a treatment and the patient just giving his consent, or with a patient selecting an alternative and the physician merely being requested to implement it (Charles et al. 1999, 658; Sandman and Munthe 2009; Stiggelbout et al. 2015, 1176)?

In contrast to all attention for the process, the literature still lacks serious attention for the *product* of SDM, i.e., the decision made at the end of the process, and of sharing it. For what, actually, does it mean for a medical decision to be shared? In the current paper, I address that question. First, I explore the concept of a shared medical decision. On the basis of several scenarios involving a decision to implement a specific medical alternative, I propose six criteria as jointly constituting the degree to which such a decision is shared. Then I relate these findings to the debate about SDM at large, discussing what is implied if SDM, next to a shared process, also requires a shared decision. I argue that shared decisions are valuable, and explain what role the concept of a shared decision can play in actual medical encounters. I conclude by mentioning several opportunities for further research into shared medical decisions.

## Sharing a medical decision

In every decision making process, several decisions can be made in a more or less shared manner. In the medical encounter, one obvious decision concerns a specific treatment to be implemented: say A, B, or C. However, this decision for a specific treatment is often preceded by another decision, viz. whether a treatment should be implemented in the first place, or whether it is better to refrain from treating and, e.g., opt for ‘watchful waiting’ (Charles et al. 1999, 656). Third, on a level ‘above’ these decisions stands a decision concerning the procedure for reaching them. This ‘meta-decision’ concerns the manner in which the patient is informed about (technical details of) treatment options, the way in which he is involved in weighing these options, and the influence of the physician on the final treatment decision.^7^ As the main focus of SDM literature is decision making about medical treatments, that will also be my focus below. In order to include decisions on a specific treatment as well as decisions on ‘treating or not treating’, I will write about decisions concerning ‘alternatives to implement’, construing the option of ‘watchful waiting’ as one of the alternatives that can be ‘implemented’.

As said above, a decision making process involving a physician and a patient can have several outcomes: no decision at all, a decision to ‘agree to disagree’, or a shared decision. The current paper is concerned with what is involved in the latter case: how should we construe the concept of a shared medical decision? One natural, straightforward answer to this question is given by Charles et al.:

[t]he test of a shared decision (as distinct from the decision-making process) is if both parties agree on the treatment option (Charles et al. 1997, 688).

Thus, we might simply say that a decision on an alternative to be implemented is shared just in case both the physician and the patient agree to implement that alternative. However, though such mutual agreement is an important aspect (and, plausibly, a necessary condition) of a shared decision, this construal does not yet accommodate the fact that mutual acceptance allows of *degrees*. Many commentators hold that SDM is a gradual concept: that decision making can be shared to a high or low degree (cf. Charles et al. 1997, 685; Makoul and Clayman 2006, 307; Zeiler 2007, 284-5).^8^ Similarly, it appears reasonable to construe the concept of a shared decision as being gradual: a decision can be minimally shared, or somewhat shared, or very shared, etc. As Charles et al. acknowledge,

[mutual acceptance] does not mean that both parties are necessarily convinced that this is the best treatment for this patient, but rather that both endorse it as the treatment to implement. The physician may feel, for example, that the patient would really be better off with another treatment but agrees to endorse the patient’s choice as part of a negotiated agreement in which the patient’s views count (1997, 688).

Thus even when the physician and the patient agree to implement a specific alternative, there is still room for much variation. The parties can fully agree that the chosen alternative is in fact the best alternative, the chosen alternative can be the favorite option of only one of the parties while the other regards it far from optimal, the decision can be a compromise where both parties have to make a concession, etc.

Taking seriously the thought that the concept of a shared decision is gradual, the question becomes how we should construe the *degree* to which a medical decision is shared: when is a decision only minimally shared, when is a decision very shared, and what conditions influence its degree of being shared?

Intuitively, and in line with the above passage from Charles et al., the degree to which a decision is shared depends on how both parties envisage the chosen alternative. If they both favor that alternative very much, their decision is shared to a high degree; yet if one party favors the alternative but the other does not, the decision is shared to a lower degree. As an illustration, consider a case where a physician diagnoses her patient with uncomplicated acute appendicitis. Given this condition, the following 5 alternatives might be contemplated:

A: removing the appendix by laparoscopic surgery, involving several small incisions in the abdomen.
B: removing the appendix by open laparotomy, involving one larger incision in the lower-right area of the abdomen.
C: using antibiotics to see if surgery can be avoided, involving a risk that surgery will later turn out necessary after all.
D: using probiotics to restore the microbial balance and suppress the growth and colonization of pathogenic microorganisms, in the hope that surgery can thereby be avoided.
E: watchful waiting to see how the appendicitis develops, relieving pain with suitable pain medication.

It is well imaginable that the physician and the patient appreciate these alternatives very differently. The physician may prefer a treatment which cures the patient as safely and efficiently as possible, favoring options A and B over the others. Possibly, the patient shares the physician’s attitude. In that case, a decision, e.g. to implement A or B, will be shared to a high degree. However, the patient may also disagree with the physician. For instance, he may dread the idea of surgery, or really dislike the prospect of a (small) skar, and prefer alternatives C and D. Or he may not like the idea of any medical procedure whatsoever, and favor alternative E. If the physician and the patient succeed in reaching an agreement in the latter cases, their decision will probably be a compromise, where one of them has (or both have) to make a concession. The decision will then be shared to a lower degree.^9^

In order to make these intuitive thoughts about a decision’s being shared a bit more precise, let us take a closer look at possible scenarios involving a physician and a patient and their preferences regarding various alternatives. As above, let us consider a case where a physician and a patient contemplate 5 alternatives: A, B, C, D, and E.^10^ And let us suppose they decide to implement alternative D. Now consider the following scenario (Scenario 1, Table 1), with orderings representing the preferences of the physician and the patient, where the alternative at the top is preferred most and the one at the bottom is preferred least.

**Table 1:** Scenario 1

Physician	Patient
B	A
**D**	E
C	**D**
A	B
E	C

Alternatively, consider the slightly different rankings in Scenario 2 (Table 2):

**Table 2:** Scenario 2

Physician	Patient
B	A
C	E
**D**	**D**
A	B
E	C

Though D is implemented in both scenarios, in a sense the degree to which the choice for D is shared is higher in Scenario 1 than in Scenario 2. In Scenario 2, D is the third option for both, whereas in Scenario 1 it is the second option for the physician and the third option only for the patient. Since in in Scenario 2, 4 alternatives are favored more (B, C, A, and E), and in Scenario 1, 3 alternatives are favored more (B, A, and E), jointly the parties favor D most in Scenario 1. Thus, a decision’s degree of being shared seems to be influenced by the ranking of the chosen alternative by both parties.^11^

We might quantify this by assigning ‘ranking scores’ to the physician’s and the patient’s preferences: 5 points for the most preferred alternative, 4 for the second, etc.; and considering the decision with the highest score to be the one shared to the highest degree. In that case, D would receive a score of 7 (4 + 3) in Scenario 1, and a score of 6 (3 + 3) in Scenario 2, which fits the intuitive, qualitative judgment made above.^12^

Though a construal in terms of rankings is natural, it should be supplemented by other considerations. The reason for this is that ranking scores may give a misleading representation of a party’s preferences. For instance, in Scenario 2, the physician may consider B the best alternative, C and D very attractive but still second and third best alternatives, and A and E far less attractive than B, C, and D. In that case, assigning ranking scores in the linear way suggested above fails to do justice to important details of the physician’s preferences.

In light of this, we may allow both parties to assign individual ‘preference scores’ to the alternatives, say varying from 1 to 5 (or represent them as having such scores); and then add the scores assigned to the chosen alternative in order to determine the degree to which the decision to implement that alternative is shared.^13^ Thus, consider the rankings including preference scores for scenarios 1 and 2 in Tables 3 and 4:

**Table 3:** Scenario 1 with preferences scores

Physician		Patient	
B	5	A	5
**D**	3	E	4
C	1	**D**	2
A	1	B	1
E	1	C	1

**Table 4:** Scenario 2 with preference scores

Physician		Patient	
B	5	A	5
C	4	E	5
**D**	4	**D**	5
A	1	B	1
E	1	C	1

The more subtle scores in these tables result in different evaluations for the alternatives: in Scenario 1, D receives a score of 5 (3 + 2), whereas in Scenario 2, D receives a score of 9 (4 + 5). Unlike the conclusion based on rankings, the preference scores suggest that the degree to which the decision to implement D is shared is higher in Scenario 2 than in Scenario 1. This outcome is intuitive: in Scenario 2, both the physician and the patient highly value D, whereas in Scenario 1, both the physician and the patient attach a lower value to D; and jointly, the parties favor D much more in Scenario 2 than in Scenario 1.

The above scenarios show a construal based on preference scores trumping a construal based on rankings. This raises the question whether the degree of a decision’s being shared should be construed exclusively in terms of preference scores or whether their rankings may still be relevant too. In order to make this issue more tangible, consider scenarios 3 and 4, again involving a decision to implement D:

(3) D is assigned a score of 4 by both parties, and D is ranked 1st by both;
(4) D is assigned a score of 4 by both parties, and D is ranked 3rd by both.

In terms of the preference scores assigned to D, the decision is shared to the same degree in (3) and (4). However, as both parties rank D third in (4) and first in (3), it seems that the situations are significantly different. In (3), the degree to which the parties jointly favor D seems higher than in (4), as neither has to make a concession in that scenario, while in (4) both favor other alternatives more. Hence, it is reasonable to regard the decision’s degree of being shared higher in (3). Similarly, consider the following two scenarios:

(5) D is assigned a score of 3 by both parties, and D is ranked 1st by both;
(6) D is assigned a score of 4 by both parties, and D is ranked 3rd by both.

In (6), the decision receives higher preference scores than in (5). Yet in (5), D is the favorite option of both parties, implying a joint preference and no need to make concessions for both parties. Hence, just as with regard to (3) and (4), it seems to make sense to consider the degree of being shared higher in (5) than in (6). So whereas in Scenario 1 and Scenario 2 rankings were trumped by preference scores, in scenarios (3) and (4) and scenarios (5) and (6) preference scores may be trumped by rankings. Apparently, determining the degree of a decision’s being shared requires consideration of both preference scores and rankings.

Next to these two considerations, several other aspects seem also relevant. Consider the following scenarios:

(7) D is assigned a score of 3 by both parties, and D is ranked 2nd by both;
(8) D is assigned a score of 4 by the physician and 2 by the patient, and D is ranked 2nd by both.

Both in terms of the preference scores assigned to D (twice 6), and in terms of the ranking scores (twice 8), the decision is shared to the same degree in (7) and (8). However, the preference scores assigned to D in (7) are the same, whereas in (8) the physician’s score is much higher than the patient’s. Thus in a sense, the decision is shared more in (7) than in (8): in (7), but not in (8), the physician and the patient are equally happy with the decision made, sharing their appraisal of D. If we take this into account, an additional consideration for determining the degree to which a decision is shared is ‘similarity of preference score’.

For an analogous fourth factor to be taken into account, consider the following two scenarios:

(9) D is assigned a score of 3 by both parties, and D is ranked 2nd by both;
(10) D is assigned a score of 3 by both parties, and D is ranked 1st by the physician and 3rd by the patient.

Both in terms of the preference scores assigned to D (twice 6), and in terms of the ranking scores (twice 8), the decision is shared to the same degree in (9) and (10). However, in (9) the physician and the patient rank D the same, whereas in (10) the physician ranks D much higher than the patient. So, again, in a sense D is shared more in (9) than in (10): in (10) the physician is happier with D than the patient, and does not have to make a concession while the patient does, whereas in (9) both parties are equally satisfied with D, sharing their appraisal of D and the amount of alternatives they favor more. If we take this into account, a fourth consideration for determining the degree to which a decision is shared is ‘similarity of ranking’.

A fifth factor to be taken into account concerns the concessions to be made by the physician and the patient. Consider the following two scenarios:

(11) D is assigned a score of 3 by both parties, D is ranked 2nd by both, and both parties assign their first alternative a score of 5;
(12) D is assigned a score of 3 by both parties, D is ranked 2nd by both, and both parties assign their first alternative a score of 4.

In terms of the preference scores (twice 6), the ranking scores (twice 8), and also in terms of the similarity of the scores and the similarity of rankings, the decision to implement D is shared to the same degree in (11) and (12). However, for both parties the difference in scores between D and the alternative to be given up is greater in (11) than in (12): 5–3 vs. 4–3. In that sense, the parties have to give up more in (11) than in (12), so that (11) involves a greater ‘total concession size’ than (12). Hence, analogous to the consideration in terms of both parties *favoring* D most, we may say that D is shared more in (12) than in (11) because in (12), jointly the parties experience *least dissatisfaction* with D. Thus, a fifth consideration for determining the degree to which a decision is shared is ‘total concession size.’^14^

For a sixth and final factor to be taken into account, consider the following two scenarios:

(13) D is assigned a score of 3 by both parties, D is ranked 2nd by both, the physician assigns her first alternative a score of 5, and the patient assigns his first alternative a score of 3;
(14) D is assigned a score of 3 by both parties, D is ranked 2nd by both, and both parties assign their first alternative a score of 4.

In terms of the preference scores (twice 6), the ranking scores (twice 8), the similarity of the scores and the similarity of rankings, and also in terms of the total concession size ([5–3] + [3–3] vs. [4–3] + [4–3]), the decision to implement D is shared to the same degree in (13) and (14). However, in (14) the physician and the patient assign the same score to the alternative they have to give up, whereas in (13) the physician assigns her first alternative a much higher score than the patient: for the patient, the difference between his first and his second option may be negligible. Thus in a sense, the decision is shared more in (14) than in (13): in (14) the physician and the patient make a similar offer, sharing their dissatisfaction, whereas in (13) the physician has to make a much bigger concession than the patient. If we take this into account, a sixth relevant consideration is ‘similarity of concession sizes’.

Given the above considerations, we may conclude that the degree to which a decision to implement an alternative is shared is influenced by six considerations: (i) how the physician and the patient rank that alternative, (ii) the preference scores the physician and the patient (would) assign to that alternative, (iii) the similarity of the preference scores, (iv) the similarity of the rankings, (v) the total concession size, and (vi) the similarity of the concession sizes.

A next question is of course how taking these six considerations into account in a *particular* case can help to establish a specific degree of a decision’s being shared. A natural thought is that the six considerations can all receive a specific score, and that the sum of those scores denotes the degree to which the decision is shared. However, this way of establishing a degree of being shared may be too simplistic, as it is imaginable that some considerations should receive more weight than others. In that case, specifying degrees of being shared would require assigning weights to the individual considerations.

Obviously, a challenge facing any attempt to establish precise degrees of being shared is to determine how scores can be attributed to the six considerations. A challenge specifically for the attempt to establish degrees of being shared using weights for the six considerations is to determine what those weights should be. Both challenges require further conceptual research, a task I leave for another occasion. At this point, I simply accept the basic rule saying that the degree to which a medical decision is shared is determined by the six considerations explicated above, such that (a) the better the decision performs in terms of those considerations, the higher its degree of being shared, (b) the decision is shared to a high degree when it performs well in terms of those considerations, and (c) the decision is shared to a low degree when it performs poorly in terms of those considerations.

## Implications: the process, the product, and the physician

Given the above analysis of a shared medical decision, a question arises as to the relation between the concept of a shared decision and the concept of SDM: does SDM require that, in addition to the decision making process being shared, the decision is shared as well? And if so, to what degree should the decision be shared? One may think that shared decision making requires that both the process and the decision are shared. After all, it appears counterintuitive to speak of ‘shared decision making’ when no shared decision has been made. While important parts of the decision making may have been shared, something still seems to be missing in that case. This intuitive thought is endorsed by several commentators. For instance, Charles et al. hold that “[m]utual acceptance (…) is a necessary prerequisite for shared decision-making” (1997, 688). For them, this ‘mutual acceptance’ only requires that the physician and the patient both accept a specific alternative; the degree to which this decision is shared may still be low (ibid., 688). Similarly, Towle and Godolphin hold that “shared decision making should lead to an agreed decision”, even when this may require discussion or negotiation (1999, 768). And, finally, Coulter holds that SDM implies that “both the *process* of decision-making and the *outcome* – the treatment decision – will be shared” (1997, 113).

If we follow these commentators in assuming that SDM requires both a shared process and a shared decision, it seems that current instruments for measuring SDM should be extended. For if SDM requires a shared decision, it is plausible that the *degree* of SDM is also influenced by the *degree* to which the decision is shared. However, current instruments do not include items concerning details of the decision. For instance, SDM-Q-9, measuring SDM from the perspective of the patient, features the following 3 items related to the decision:

7. My doctor and I thoroughly weighed the different treatment options.
8. My doctor and I selected a treatment option together.
9. My doctor and I reached an agreement on how to proceed (Kriston et al. 2010, 98).

Analogously, SDM-Q-Doc, measuring SDM from the perspective of the physician, has the following 3 items indirectly related to the decision:

7. My patient and I thoroughly weighed the different treatment options.
8. My patient and I selected a treatment option together.
9. My patient and I reached an agreement on how to proceed (Scholl et al. 2012, 288).

Finally, OPTION-5, a ‘measure of shared decision making’, only features the following item related to the decision:

5. Integrate preferences. The provider makes an effort to integrate the patient’s preferences as decisions are either made by the patient or arrived at by a process of collaboration and discussion (Elwyn et al. 2013, 269).

These instruments all address the deliberation about alternatives, and also the selection of an alternative and its implementation. However, none of them seriously relates to the degree to which the decision is shared, for instance by addressing how the patient or the physician evaluates the selected alternative.^15^

In order to measure not only the degree to which the process is shared but also the degree to which the decision is shared, SDM measures should ideally accommodate the six criteria from the previous section, which would require items concerning (a) the ranking of the selected alternative in relation to alternatives favored more, and (b) the preference scores assigned to the selected alternative in comparison to alternatives favored more. Moreover, given that the six criteria involve comparisons between rankings and scores by the physician and the patient, measuring the degree to which a *specific* case of decision making is shared would require consideration of rankings and scores by the physician and the patient with regard to the decision made in that specific case. If acquiring all this information turns out to be unrealistic, a less ambitious proposal would be to focus only on a subset of the considerations, e.g. by including only items with regard to the selected alternative, so that at least some considerations, e.g. (ii) and (iii), can be taken into account.

Another issue involved by adding items with regard to the decision to SDM measures concerns their relation to the items regarding the process. For instance, suppose that 2 decision related items are added to SDM-Q-9 (and SDM-Q-Doc). What should be their influence on the degree of SDM as such? Should these 2 items carry the same weight as the other 9? Or should the scores on the decision items and the scores on the process items constitute two separate parts of the total SDM score, such that the latter is determined by adding the averages of the decision scores and the process scores, or by adding these averages corrected by weights corresponding to the relative importance of the process and the product?

Given these challenges implied by the assumption that SDM requires a shared process *and* a shared decision, we might also deny the assumption, and settle for SDM as merely a shared process. This option is suggested by Makoul and Clayman:

[W]hile it has been suggested that a mutually agreed upon course of action is the appropriate result of SDM, a difference of opinion between physician and patient may still exist at the end of the SDM process. We recognize that mutual agreement is highlighted in each of the prominent models [of SDM], but believe it is properly positioned as an ideal rather than a necessity (Makoul and Clayman 2006, 306).

In line with denying that SDM requires a shared decision, one could further downplay the relevance of shared decisions as compared with shared processes. After all, one might argue, is the reason that SDM has gained widespread acceptance not mainly the value attached to the process of the physician and the patient exchanging information, deliberating, and attempting to reach a decision, rather than the value associated with a shared product? And is not the most important function of SDM that the two collaborate well, their decision making contrasting with traditional paternalism and the information model?

In response to these questions, several things should be noticed. First of all, even if a decision’s being shared is less relevant with regard to SDM, the concept of a shared decision seems valuable for other reasons as well. Intuitively, a situation where a physician and a patient both favor a selected alternative (or favor that alternative in a similar way) is preferable to a situation where one of them is much less happy with that alternative. Moreover, it may well be that highly shared decisions, as contrasted with moderately shared decisions, have beneficial effects comparable to those associated with highly shared decision making processes (e.g. Elwyn et al. 2012, 1362; Kashaf and McGill 2015; Stacey et al. 2017). For example, it is imaginable that shared decisions positively contribute to patient compliance, patient satisfaction, patient wellbeing, patient confidence in decisions, etc.^16^

Finally, and most importantly, there are also SDM related reasons for appreciating shared decisions. These reasons concern two important motivations underlying the ideal of SDM: avoiding paternalism and respecting patient autonomy (cf. Emanuel and Emanuel 1992; Sandman and Munthe 2009; Lewis 2019). As is documented in the literature, preferences regarding roles in medical decision making are mixed.^17^ Some patients prefer a passive role. For instance, Stiggelbout et al. (1997) found that especially older patients and men are more likely to prefer not to be actively involved in decision making; and Levinson et al. (2005) found that men, less educated patients, and unhealthier patients are more likely to prefer a passive role. At the same time, though, most studies report that the majority of patients do want to participate in decision making: they want to share relevant information, or deliberate about treatment options, or be involved in the final decision (e.g. Stiggelbout et al. 1997; Charles et al. 1998; Levinson et al. 2005; Chewning et al. 2012).

If a patient prefers a passive role, and asks the physician to make the final decision, what would respecting autonomy require of the physician? One thought, sometimes expressed in the literature, is that she should attempt to *encourage* the patient to use, or develop, his autonomy: being a mature, competent human being, it should be possible to inspire him to participate in sharing information, deliberating, and deciding (e.g. Stiggelbout et al. 1997, 388; Levinson et al. 2005, 534). Presumably, though, if it turns out difficult or impossible to let the patient partake in decision making, and he keeps insisting that the physician makes the decision, then respecting autonomy may well be thought to require honoring this wish, e.g., as a sincere choice to trust the physician’s expertise (cf. Levinson et al. 2005, 534). Despite the putative benefits of sharing decision making, if the physician wants to respect patient autonomy, she should then individually make a decision based on all the information available to her, ideally including relevant information about the patient’s perspective.^18^

However, if a patient wants to be actively involved in the decision making process, and also wants to have a say in the final decision, respecting autonomy seems to demand a different attitude. In that case, it requires that the patient is allowed to participate in the process, and given the opportunity to provide information, to deliberate about alternatives, and to partake in the final decision. Moreover, it also requires that the final *decision* is shared, at least to some degree. To see why, imagine a scenario where the patient states that he wants to participate in decision making, where the unfolding decision making process is highly shared in terms of information sharing, deliberation, etc., but where the decision finally made is the alternative favored most by the physician and least by the patient. In that case, the decision, though resulting from a shared process, is a paternalistic outcome which fails to honor the patient’s ability and desire to govern his own life.^19^ Certainly, if avoiding paternalism and respecting autonomy support the ideal of SDM and shared decision making *processes*, the same principles would equally well justify an ideal of shared decisions, at least with patients who wish to be actively involved in decision making.^20^

Naturally, the above conclusions have implications for the interaction of physicians with their patients in actual clinical encounters. If a physician attempts to reach a shared decision with her patient (because she wants to work in accord with SDM, or because of the putative benefits of shared decisions, or because she wants to respect patient autonomy), the analysis in this paper implies that she has to find out whether choosing a specific alternative would result in a shared decision. That is, she has to find out, at least in some detail, how her patient ranks the various alternatives under consideration, so that she can compare his ranking with her own, in particular in terms of the six considerations identified in the previous section. It may be that, analogous to challenges for measuring shared decisions, asking patients for their rankings in a formal way is unrealistic or otherwise undesirable. In that case, a physician might still be able, on the basis of her discussion with the patient, to get a sense of how his preferences are roughly organized, and determine on the basis of his preferences and her own which alternative would probably lead to a highly or sufficiently shared decision.

However, even when a physician manages to figure out which alternative would lead to a shared decision, the clinical situation may still include other values competing with that of a shared decision. For instance, proposing a specific alternative may lead to a shared decision while at the same time being suboptimal in light of a patient’s health, or survival, or in light of other values such as social justice or sustainability. Thus, a highly shared decision need not be a morally right decision. In cases where values conflict in this way, a physician faces a challenge to weigh the value of a shared decision against those other values. It is her responsibility to select an alternative that would be morally best, all things considered.

## Conclusion

In this paper I have addressed the concept of a shared decision: the product of a decision making process. Focussing on decisions to implement a specific medical alternative (possibly the alternative of ‘no treatment’), I found that the degree to which such a decision is shared should be determined by taking into account six considerations: (i) how the physician and the patient rank that alternative, (ii) the preference scores the physician and the patient (would) assign to that alternative, (iii) the similarity of the preference scores, (iv) the similarity of the rankings, (v) the total concession size, and (vi) the similarity of the concession sizes. I have sketched implications of assuming that SDM requires a shared process and a shared decision, argued for the value of shared decisions, and mentioned the implications of the analysis for practicing physicians.

The findings of this paper add to existing literature, but they also generate new questions. As we saw, one question is how the degree to which a decision is shared can be measured. Further research may be devoted to developing tools for doing so. Other questions concern the relationship between the process and the product of decision making. If SDM requires both a shared process and a shared product, existing SDM measures should be extended with items corresponding to the decision made. Regardless of whether SDM requires both a shared process and a shared product or only a shared process (or product), it would be interesting to investigate the relationship between the two, for instance to see whether a highly shared process results in a highly shared decision more often than a moderately shared process; whether sharing more specific details about treatment alternatives affects the degree of a decision’s being shared; whether a deliberation including the exchange of arguments influences the degree of a decision’s being shared; etc. And as suggested, analogous to research into possibly beneficial effects of the process of SDM, it is worth investigating what effects a highly shared decision has on, e.g., patient compliance, patient satisfaction, patient wellbeing, and patient confidence in decisions.

Finally, important questions arise about the ‘makers’ of medical decisions. While I have assumed clinical encounters featuring a patient and a physician, treatment decisions are often made by a patient and a large medical team including several care professionals. It may be thought that modelling the degree to which a decision is shared in scenarios involving more than one professional requires taking into account the preferences of all of them. However, how this should be done is a question in need of further reflection. Obviously, not the preferences of all professionals directly and indirectly related to the situation should be considered. Yet, how can a distinction be drawn between sufficiently relevant and irrelevant professionals? Should the preferences of all *relevant* professionals carry the same weight, or should the preferences of some be regarded more important? And who should decide about these issues?

Moreover, just as in the discussion in the previous section concerning the individual physician, a question arises here about the moral status of an interprofessionally shared decision. As explained, a clinical situation may include other values competing with that of a shared decision. Given a specific method to accommodate the professionals’ preferences, it may even be the case, especially when there are relatively many professionals involved, that the decision most highly shared by all decision makers in the situation fails to respect patient autonomy. Such scenarios demonstrate that a highly shared decision need not be a morally right decision. How the relation between the value of shared decisions and other values should be conceptualised is a question which deservers further investigation.

Next to the amount of caregivers, there are situations where a decision on an alternative has to be made by a physician (or medical team) and more than one patient. As illustrated by Zeiler (2007), the latter can happen in the context of new reproductive medicine, where a physician often has to reach a decision which directly involves at least two persons. In such cases the degree to which a decision is shared is influenced by the preferences of both patients. Furthermore, even when a decision involves only one patient in a direct way, his perspective may still be informed, or even constituted, by more than one individual. As has been argued by Van Nistelrooij et al. (2017), when a patient’s important relatives express their opinions about clinical alternatives, these need not merely be the views of ‘invaders’, or external ‘threats’. Rather, their views may partly *constitute* the identity of the patient. If that is so, questions emerge with regard to the relevance of these relatives’ preferences. For instance, does construing the degree to which a decision is shared also require accommodating them? If so, how should their preferences be weighted as compared to the preferences of the patient? And, assuming that not all imaginable relatives deserve to be taken into account, how can a distinction be drawn between significant and insignificant relatives?

Clearly, both the amount of questions arising, and their complexity, point to the importance of further research into shared medical decisions.

## Footnotes

1 For others recognizing the distinction between a shared process and a shared decision, see Lewis (2020, 120); Sandman et al. (2012, 126); and Coulter (1997, 113). The distinction between the decision making process and its product should not be conflated with a related distinction, drawn by Edwards and Elwyn (2006), between “the process of involvement (option portrayal, exchange of information, and exploring preferences for who makes the decision and when) and the actual decisional responsibility (who makes the decision)” (315; cf. Lewis 2020, 119 − 20). Edwards and Elwyn’s reference to ‘the process of involvement’ resembles what I regard as the decision making process, but ‘decisional responsibility’ or ‘who makes the decision’ is conceptually distinct from the decision finally made.

2 For others writing about SDM as a ‘process’, see Clayman et al. (2012, 371); Sandman et al. (2012, 120); Gillick (2015, 785); Van Nistelrooij et al. (2017, 639); Pieterse et al. (2018, 1507); and Michalsen et al. (2019, 1261).

3 For others talking about shared ‘decisions’, see Towle and Godolphin (1999, 768); Chewning et al. (2012); and Elwyn et al. (2012, 1561).

4 Zeiler, in wondering whether SDM requires only a shared process or also a shared decision, also assumes that the sharedness of the decision is not just a function of the sharedness of the process (2007, 283).

5 For other categorizations of the steps in an SDM process, see e.g. Elwyn et al. (2012) and Stiggelbout et al. (2015).

6 When writing about ‘a physician’ and ‘a patient’, I refer to the physician with ‘she’ and to the patient with ‘he’.

7 If a patient prefers not to be actively involved in the decision making process, and the physician respects this preference, that may have consequences for the sense in which their decision making will be shared. I come back to this scenario later on. Next to the three decisions mentioned in the main text, a decision making process also involves several decisions which are made individually and cannot reasonably be expected to be shared. For instance, a physician has to decide which information about the disease and about available treatments she is going to share with the patient (cf. Wirtz et al. 2006, 119–121), and the patient has to decide which personal information he will share. These individual decisions are not relevant for the purposes of the current paper. For more on decisions made in a physician-patient encounter, see Ofstad et al. (2016), whose taxonomy of medical decisions in hospital encounters features 10 categories.

8 Sandman and Munthe’s (2010) inventory of 9 versions of SDM, ranging from versions which show very little respect for patient or physician autonomy to versions which involve very much respect for it, implicitly endorses this view.

9 For discussion of another example including preferences regarding several reasonable treatment alternatives, see Charles et al. (1998), whose study focuses on women with early stage breast cancer and the effect of their preferences on the decision making process.

10 The explication below does not depend on this choice for 5 alternatives. A similar analysis can be made with scenarios involving more or less alternatives.

11 Gustavsson and Sandman (2015) also mention orderings or ‘rankings’ by physicians and patients, where physician orderings are mainly based on clinical evidence and ‘objective’ patient needs, while patient orderings might also be informed by desires (20–21). Gustavsson and Sandman do not further elaborate on the details and implications of these orderings, however. Similarly, Sandman et al. (2012) write about a ‘preference set’ of the patient (not of the physician), yet without going into the details and implications of such a set vis-à-vis SDM (120-1). The current paper can be seen as supplementing these analyses.

12 This way of conceptualizing alternatives and preferences about them resembles discussions in social choice theory. One of the questions addressed in that field is how individual judgments or preferences can be aggregated into a collective judgment or preference in a way that satisfies several rationality conditions (e.g. Arrow 1951/1963; Craven 1992; List 2022). The project of the current paper is different. Most notably, I do not consider whether decisions satisfy rationality conditions, but to what degree they are *shared* (which they may be to a high degree even if they do not satisfy all relevant rationality conditions).

13 In the language of social choice theory, this constitutes a shift from ‘ordinal preferences’ (or utilities) to ‘cardinal preferences’ (cf. e.g. Craven 1992, 134-5).

14 I assume that in cases such as (11) and (12), where D is the second alternative for both parties, the most relevant contribution to ‘concession size’ is the difference in evaluation between D and the alternative that has to be given up. This difference seems more relevant than, e.g., the scores assigned to the alternative given up. For instance, if one would assign a score of 5 both to one’s favourite alternative and to D, the concession one would have to make is much smaller than if one would assign a score of 5 to one’s favourite alternative and a score of 3 to D. The situation is more complex when (at least) one party prefers more than one alternative to D. With which scores should the score of D then be compared: with the score of the alternative ranked first, or with the score of the alternative ranked directly above D, or with the average of the scores of alternatives ranked above D? I leave discussion of this question for another occasion.

15 Another instrument ignoring the degree to which a decision is shared is MAPPIN’SDM (Kasper et al. 2012, 4).

16 It has been suggested to me by Maud Jonker that when an alternative is implemented which the physician values much higher than the patient (so that the decision is little shared), but the implementation has a suboptimal or bad clinical outcome, there might be a higher chance that the patient will blame the physician or file a complaint than in a scenario involving a highly shared decision (cf. Charles et al. 1998, 85). Of course, whether shared decisions have this or any of the other mentioned effects is a question in need of further research.

17 I thank an anonymous referee for the suggestion to relate this fact to the discussion of shared decisions.

18 A question with regard to this scenario is whether the final decision will be shared. Given the patient’s preference to delegate, one might think the decision must be unshared or even paternalistic. However, in light of the analysis from the previous section, it may also be argued that the decision will be (highly) shared, in particular if the patient’s choice to delegate the decision is taken to imply that his preferences are *identical* to those of the physician. If the physician chooses to implement her most preferred alternative, the decision might then even be maximally shared (and *will* be maximally shared if she also assigns the highest possible preference score to that alternative). In that case, not only honouring the patient’s wish to delegate decision making would be autonomy respecting, but also the final decision itself would respect patient autonomy.

19 When the alternatives under consideration, including the alternative preferred by the patient, have comparable medical prospects, this paternalism is often regarded undesirable. However, there might also be cases where a paternalistic decision is regarded justified. Suppose a patient’s most preferred alternative(s) would cause serious harm to the patient. For example, think of a Jehovah’s Witness who prefers to receive no blood transfusion for himself or his child when this is medically necessary. In such a case, a paternalistic decision, though not shared, may still be considered morally justified or even mandatory. Assuming that paternalistic decisions can be justified in such situations, the principle of avoiding paternalism only supports the value of shared decisions in the more common cases where patients are seen to have medically reasonable preferences. Analogous to scenarios involving a paternalistic decision, one can also imagine situations where, following a shared process, the decision is the alternative favoured most by the patient and least by the physician. In that case, the decision, though resulting from a shared process, and avoiding paternalism and respecting patient autonomy, would fail to respect the *physician’s* autonomy. Assuming that her autonomy is just as valuable as that of the patient, physician autonomy is also a reason to value shared decisions. I thank two anonymous referees for letting me think about concrete cases of paternalistic decisions following upon shared processes.

20 A stronger claim would be that there is *more* justification for an ideal of shared decisions than for an ideal of shared processes because unshared decisions are *worse* than unshared processes: perhaps autonomy is better respected with an unshared process but a shared decision than with a shared process but an unshared decision. I leave this a suggestion for further investigation.
